# Expression of ADAM15 in rheumatoid synovium: up-regulation by vascular endothelial growth factor and possible implications for angiogenesis

**DOI:** 10.1186/ar1796

**Published:** 2005-08-05

**Authors:** Koichiro Komiya, Hiroyuki Enomoto, Isao Inoki, Satoko Okazaki, Yoshinari Fujita, Eiji Ikeda, Eiko Ohuchi, Hideo Matsumoto, Yoshiaki Toyama, Yasunori Okada

**Affiliations:** 1 Department of Pathology, School of Medicine, Keio University, Shinjuku-ku, Tokyo, Japan; 2 Department of Orthopaedic Surgery, School of Medicine, Keio University, Shinjuku-ku, Tokyo, Japan; 3 Biopharmaceutical Department, Daiichi Fine Chemical Co. Ltd., Takaoka, Toyama, Japan

## Abstract

ADAMs (a disintegrin and metalloproteinases) comprise a new gene family of metalloproteinases, and may play roles in cell-cell interaction, cell migration, signal transduction, shedding of membrane-anchored proteins and degradation of extracellular matrix. We screened the mRNA expression of 10 different ADAMs with a putative metalloproteinase motif in synovial tissues from patients with rheumatoid arthritis (RA) or osteoarthritis (OA). Reverse transcription PCR and real-time quantitative PCR analyses indicated that among the ADAMs, ADAM15 mRNA was more frequently expressed in the RA samples and its expression level was significantly 3.8-fold higher in RA than in OA (p < 0.01). *In situ *hybridization, immunohistochemistry and immunoblotting demonstrated that ADAM15 is expressed in active and precursor forms in the synovial lining cells, endothelial cells of blood vessels and macrophage-like cells in the sublining layer of RA synovium. There was a direct correlation between ADAM15 mRNA expression levels and vascular density in the synovial tissues (*r *= 0.907, p < 0.001; n = 20). ADAM15 was constitutively expressed in RA synovial fibroblasts and human umbilical vein endothelial cells (HUVECs), and the expression level was increased in HUVECs by treatment with vascular endothelial growth factor (VEGF)_165_. On the other hand, ADAM15 expression in RA synovial fibroblasts was enhanced with VEGF_165 _only if vascular endothelial growth factor receptor (VEGFR)-2 expression was induced by treatment with tumor necrosis factor-α, and the expression was blocked with SU1498, a specific inhibitor of VEGFR-2. These data demonstrate that ADAM15 is overexpressed in RA synovium and its expression is up-regulated by the action of VEGF_165 _through VEGFR-2, and suggest the possibility that ADAM15 is involved in angiogenesis in RA synovium.

## Introduction

In rheumatoid arthritis (RA), the affected joints develop chronic synovitis that is characterized by hyperplasia of lining cells, infiltration of inflammatory cells and abundant neovascularization. Various factors such as proteinases, growth factors and cytokines are produced in the RA synovium and implicated in the destruction of articular cartilage and subchondral bones, leading to disability of the joints. Among the proteinases, matrix metalloproteinases (MMPs), a gene family of zinc metalloproteinases, are well known to play a major role in the proteolytic degradation of extracellular matrix (ECM) macromolecules of cartilage and bone, which is a key step in joint destruction in RA [1]. Members of a new family of metalloproteinases, the 'a disintegrin and metalloproteinases' (ADAMs), which share structural homology with MMPs and snake venom metalloproteinases, have recently been cloned. ADAMs consist of propeptide, metalloproteinase, disintegrin-like, cysteine-rich, epidermal growth factor-like, transmembrane and cytoplasmic tail domains [1,2]. Members are classified into putative proteinase-type and non-proteinase-type ADAMs according to the different structures of the catalytic site motif in the metalloproteinase domain [1,3]. Although the specific biological functions of ADAMs are not well elucidated at the present time, they may be involved in cell-cell interaction, cell migration, signal transduction, shedding of various membrane-anchored proteins and degradation of ECM components under pathophysiological conditions such as fertilization [4,5], morphogenesis [6,7], angiogenesis [8] and cancer [9]. The expression of ADAM10, ADAM15 and ADAM17 in arthritic cartilage and synovial tissues has been examined [10-12], but there are no reports of systematic analyses of the expression of ADAMs in arthritic joint tissues. In addition, little or no information is available for correlation between the expression and synovial pathology or for regulation mechanism of ADAM expression. Angiogenesis in the synovium during RA begins at the early stage of the disease and is crucial for progression of the synovitis [13]. Vascular endothelial growth factor (VEGF), which has five different isoforms (VEGF_121_, VEGF_145_, VEGF_165_, VEGF_189 _and VEGF_206_) is known to play a key role in the angiogenesis in RA synovium [13,14]. All these VEGF isoforms bind to high-affinity receptors, namely VEGFR-1 (fms-like tyrosine kinase; Flt-1) and VEGFR-2 (kinase insert domain-containing receptor; KDR). Neuropilin-1, an isoform-specific co-receptor of VEGFR-2, enhances the bioactivity of VEGF_165 _by increasing its binding affinity for VEGFR-2 [15]. Interestingly, binding of VEGF to its receptors on endothelial cells enhances not only their proliferation and migration but also production of MMPs [16-18]. In addition, VEGF stimulates other cells such as chondrocytes to induce expression of MMPs [19]. Thus, it might be possible to speculate that VEGF regulates the expression of proteinase-type ADAMs.

In the present study, we examined the expression of 10 different ADAM species with a putative metalloproteinase motif in synovial tissues of RA and osteoarthritis (OA), correlation of ADAM15 expression with synovial pathology, localization of ADAM15 in RA synovium, and the mechanism of regulation of ADAM15 expression in RA synovial fibroblasts and endothelial cells. Our results demonstrate that ADAM15 is expressed in lining cells, endothelial cells of blood vessels and macrophage-like cells in the sublining layer of RA synovium with a direct correlation with vascular density in the synovium, and that the expression of ADAM15 is up-regulated by the action of VEGF_165 _via VEGFR-2.

## Materials and methods

### Clinical samples and histology

Synovial tissues were obtained from patients with RA (56 ± 14 years old (mean ± SD); n = 16) or OA (73 ± 6 years old; n = 20) at total knee arthroplasty. Diagnosis of the patients with RA or OA was based on the 1987 revised American Rheumatism Association Criteria for RA [20] and the American Rheumatism Association Criteria for OA [21]. Synovial specimens were fixed with periodate-lysine-paraformaldehyde, and paraffin sections stained with hematoxylin and eosin were analyzed by light microscopy according to our grading system of synovial lining cell hyperplasia, cellular infiltration and fibrosis [22]. For the experimental use of the surgical specimens, written informed consent was obtained from the patients according to the hospital ethical guidelines.

### Reverse transcription-PCR

Total RNA was extracted directly from RA (n = 16) and OA (n = 20) synovial tissues and evaluated by using the Agilent 2100 Bioanalyzer (Agilent Technologies, Palo Alto, CA, USA) as descried previously [9]. By using a random hexamer of oligonucleotides (Takara Bio Inc., Otsu, Japan), cDNAs were prepared from total RNA with SuperScript II reverse transcriptase (Life Technologies Inc., Rockville, MD, USA). The reaction product was subjected to reverse transcription (RT)-PCR analysis on the expression of ADAMs 8, 9, 10, 12, 15, 17, 20, 21, 28 and 30, VEGFR-1, VEGFR-2, neuropilin-1 and β-actin for 25–30 cycles. PCR was carried out in 50 μl reaction volume containing 800 nM of each primer, 220 μM of dNTPs and 1 unit of ExTaq DNA polymerase (Takara Bio Inc.). The thermal cycle was 1 minute at 94°C, 1 minute at 62°C for ADAMs 8, 9, 10, 12, 17, 20, 21, 28 and 30, 67°C for ADAM15, 64°C for VEGFR-1, 63°C for VEGFR-2 and neuropilin-1 and 65°C for β-actin, and 1 minute at 72°C, followed by 3 minutes at 72°C for the final extension. The nucleotide sequences of the PCR primers and the expected sizes of the amplified cDNA fragments are shown in Table 1. Aliquots of the PCR products were electrophoresed in 2% agarose gels, and stained with ethidium bromide. For positive controls, total RNA was extracted from cancer cell lines as described previously [9]. The specific amplification of these ADAMs, VEGFRs, neuropilin-1 and β-actin was confirmed by direct sequencing of the PCR products.

### Real-time quantitative PCR for ADAM15

The mRNA expression levels of ADAM15 were evaluated in a TaqMan real-time PCR assay using the ABI Prism 7000 sequence detection system (Applied Biosystems, Foster City, CA, USA) according to the manufacturer's protocols. Cycling conditions were 50°C for 2 minutes, 95°C for 10 minutes, and then 40 cycles at 95°C for 15 seconds and 60°C for 1 minute. Primers were designed and selected using Primer Express software (Applied Biosystems). Sequences of the primers and TaqMan probe for ADAM15 were as follows: forward primer, 5'-GGCAATCGAGGCAGCAAAT-3'; reverse primer, 5'-TGGTGGAGATCAGCCCAAAC-3'; and TaqMan probe, 5'-FAM-CAGCTGTCACCCTCGAAAACTTCCTCC-TAMRA-3'. The relative quantification value of ADAM15 was normalized to an endogenous control, 18S ribosomal RNA, after confirming that ADAM15 and ribosomal 18S cDNAs were amplified with the same efficiency according to the manufacturer's protocol. The total gene specificity of the nucleotide sequences chosen for the primers and probe and the absence of DNA polymorphisms were ascertained using BLASTN and Entrez from the National Center for Biotechnology Information web site [23].

### *In situ *hybridization

Paraffin sections of the RA synovial tissues (n = 5) were used for *in situ *hybridization of ADAM15 according to a modification of our methods used previously [19]. Briefly, the sections were treated with proteinase K (5 μg/ml; Sigma-Aldrich Inc., St Louis, MO, USA) in 10 mM Tris-HCl buffer, pH 8.0, and 1 mM ethylenediaminetetraacetic acid, and endogenous peroxidase was blocked with 0.3% hydrogen peroxide in methanol. Single-stranded sense and anti-sense digoxigenin-labeled RNA probes were generated by *in vitro *transcription from the cDNA encoding ADAM15, nucleotides 1091 to 1331 (241 bp), using the DIG RNA labeling kit (Roche Diagnostics GmbH, Mannheim, Germany). BLASTN searches were performed to confirm the specificity of the probes. Hybridization with the probes was performed at 42°C for 16 h, and the sections were washed in 2× standard saline citrate/50% formamide, followed by digestion with 10 μg/ml ribonuclease A (Wako Pure Chemical Industries, Osaka, Japan). After washing once in 2× standard saline citrate and twice in 0.2× standard saline citrate and blocking nonspecific binding with blocking solution (DakoCytomation Norden A/S, Glostrup, Denmark), they were incubated with mouse anti-digoxigenin antibody (1/750 dilution; Roche Diagnostics GmbH), and subjected to the following steps using the Catalyzed Signal Amplification System (DakoCytomation Norden A/S) according to the manufacturer's protocol. Counterstaining was performed with hematoxylin.

### Characterization of monoclonal antibody against human ADAM15 and immunoblotting

**Figure 1:**
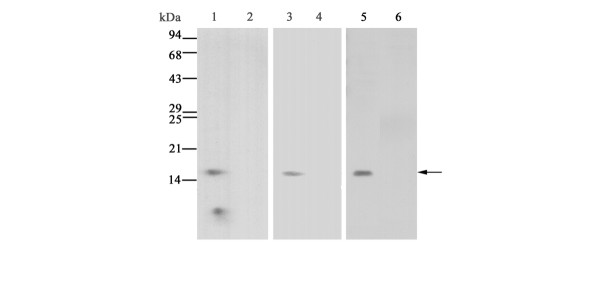
Characterization of monoclonal antibody against human ADAM15. Lysates of *Escherichia coli *transfected with the expression vector FLAG-ADAM15 containing a cDNA fragment encoding the cysteine-rich domain of human ADAM15 (lanes 1, 3, 5 and 6) or the pFLAG-CTC vector alone (lanes 2 and 4) were immunoblotted with anti-FLAG antibody (lanes 1 and 2) or anti-ADAM15 antibody (240-2C7) (lanes 3-6) as described in Materials and methods. The absorption study was carried out by incubation of the antibody with 1000-fold excess amounts of the peptide for 16 h at 4°C (lane 6). The arrow indicates the protein band of the cysteine-rich domain reactive with anti-ADAM15 antibody. Note that no staining is present with mock transfectants (lanes 2 and 4), and that the immunoreactive band with anti-ADAM15 antibody (lane 5) completely disappears after reaction with the antibody absorbed with the peptide (lane 6).

A monoclonal antibody against human ADAM15 was developed using the synthetic peptide corresponding to the amino acid sequence of the cysteine-rich domain (residues 596 to 612, RDLLWETIDVNGTELNC) of human ADAM15 as an antigen according to our methods [24]. Clones were screened by enzyme-linked immunosorbent assay using the peptide, and a clone 240-2C7 was selected as a candidate. The specific reactivity of the antibody was further evaluated by immunoblotting. The cysteine-rich domain of ADAM15 with FLAG-tag was expressed in *Escherichia coli *DH5α (Takara Bio Inc.) by transfecting with the expression vector pFLAG-ADAM15, which was prepared by inserting a cDNA fragment encoding the human ADAM15 cysteine-rich domain (nucleotides 1887 to 1937) [25] into pFLAG-CTC vector (Sigma-Aldrich Inc.). As a negative control, the vector pFLAG-CTC alone was transfected to DH5α (mock transfectants). Cell lysates were subjected to SDS-PAGE (15% total acrylamide) under reducing conditions. The resolved proteins were then transferred to polyvinylidene difluoride membranes (ATTO, Tokyo, Japan), which were reacted with anti-FLAG antibody (1 μg/ml; Sigma-Aldrich, Inc.), anti-ADAM15 antibody (1 μg/ml; 240-2C7) or non-immune mouse immunoglobulin G (IgG) (1 μg/ml; DakoCytomation Norden A/S) after blocking with 5% skim milk. They were then incubated with horseradish peroxidase-labeled anti-mouse IgG (1/5000 dilution; Amersham Biosciences Corp., Piscataway, NJ, USA). Immunoreactive bands were detected with ECL Western blotting reagents (Amersham Biosciences Corp.). As shown in Fig. 1, two protein bands of 15 kDa and 10 kDa were detected with anti-FLAG antibody in the cell lysates of ADAM15 transfectants (lane 1) but not mock transfectants (lane 2). On the other hand, anti-ADAM15 antibody (240-2C7) selectively reacted with the band of 15 kDa in the ADAM15 transfectants (Fig. 1, lane 3) but not mock transfectants (lane 4). The molecular weight of the immunoreactive 15-kDa band corresponds to that of the cysteine-rich domain of ADAM15 [25]. Importantly, the immunoreactivity of the 15-kDa band was blocked after absorption of the antibody with the antigen peptide (Fig. 1, lanes 5 and 6). Blotting with non-immune mouse IgG showed no reactive bands (data not shown). This indicates that the monoclonal antibody (240-2C7) is monospecific to the cysteine-rich domain of ADAM15.

RA synovial tissues (n = 5) were homogenized on ice in a lysis buffer (50 mM Tris-HCl buffer, pH 7.5, 150 mM NaCl, 10 mM CaCl_2 _and 0.05% Brij35) containing a cocktail of proteinase inhibitors (Roche Diagnostics, GmbH). Supernatants of the homogenates were subjected to SDS-PAGE (10% total acrylamide) under reducing conditions, transferred onto polyvinylidene difluoride membranes and reacted with anti-ADAM15 antibody (240-2C7; 1 μg/ml) or non-immune mouse IgG (1 μg/ml) after blocking with 5% skim milk. They were then incubated with horseradish peroxidase-labeled anti-mouse IgG and immunoreactive bands were detected with ECL Western blotting reagents as described above.

### Immunohistochemistry

Paraffin sections of the RA synovial samples were treated with 0.3% H_2_O_2 _and 10% normal goat serum to block endogenous peroxidase and non-specific binding, respectively. As antigen retrieval, the sections were subjected to microwave treatment at 500 W for 10 minutes in 10 mM citrate buffer, pH 6.0. They were then treated with mouse anti-ADAM15 antibody (240-2C7; 20 μg/ml), rabbit anti-VEGFR-2 antibody (Flk-1; 5 μg/ml; Santa Cruz Biotechnology, Santa Cruz, CA, USA), rabbit anti-von Willebrand factor (vWF; 15 μg/ml; DakoCytomation Norden A/S) or mouse anti-CD31 antibody (8 mg/ml; DakoCytomation Norden A/S). After the reaction with goat anti-mouse IgG or goat anti-rabbit IgG conjugated to peroxidase-labeled dextran polymer (no dilution; En Vison+ Mouse or En Vison+ Rabbit; DakoCytomation Norden A/S), the color was developed with 3,3'-diaminobenzidine tetrahydrochloride in 50 mM Tris-HCl buffer, pH 7.6, containing 0.006% H_2_O_2_. Counterstaining was performed with hematoxylin. As for a control, sections were reacted by replacing the first antibodies with non-immune mouse IgG or rabbit IgG.

### Vascular density

Vascular density in RA and OA synovial tissues was evaluated by the morphometric analysis of RA and OA tissue sections immunostained with anti-CD31 antibody without any clinical information on each sample. Four fields were selected at random and vessels with a distinct lumen were counted to calculate the number of vessels per square millimeter as we described previously [14]. The average vascular density (vessels/mm^2^) from the fields for each patient was processed for further statistical analysis.

### Cell cultures of rheumatoid arthritis synovial fibroblasts and endothelial cells

RA synovial fibroblasts (SFs) were prepared from RA synovial tissues obtained at total knee arthroplasty. The tissues were minced and incubated with 0.04% bacterial collagenase type I (Worthington Biochemical Corp., Freehold, NJ, USA). Isolated cells were seeded in culture dishes and maintained in DMEM (Life Technologies) supplemented with 10% fetal bovine serum (Life Technologies) at 37°C in humidified 5% CO_2 _in air. After the cells were cultured in confluence, they were trypsinized and reseeded in culture dishes. RA SFs at 5–9 passages were used for experiments. Human umbilical vein endothelial cells (HUVECs 7943; Cambrex Co., East Rutherford, NJ, USA) were grown in medium EBM-2 supplemented with EGM-2 (Cambrex Co.).

In order to examine the expression of VEGFR-2 and ADAM15 and exclude the possibility of contamination of cultured RA SF by endothelial cells, RA SFs at 5–9 passages were seeded on Lab-Tek II chamber slides (Nalge-Nunc International, Naperville, IL, USA) and subjected to immunohistochemistry for VEGFR-2, ADAM15, CD31 and vWF as described above. For a positive control, HUVECs were immunostained with these antibodies. In addition, mRNA expression of CD31 and vWF in cultured RA SFs was examined by RT-PCR using the PCR primers (Table 1).

### Stimulation of RA synovial fibroblasts with proinflammatory cytokines and/or growth factors

RA SFs were plated on a 60 mm dish at a density of 3 × 10^5 ^cells/dish in DMEM supplemented with 10% fetal bovine serum. The culture media were replaced with serum-free DMEM containing 0.2% lactalbumin hydrolysate and starved for 24 h before they were used in experiments. The cells were treated with tumor necrosis factor-α (TNF-α; Dainippon Pharmatheutical, Osaka, Japan), IL-1α (Dainippon Pharmatheutical), transforming growth factor-β (TGF-β; R&D Systems, Minneapolis, MN, USA; 0, 0.1, 1 or 10 ng/ml) or recombinant VEGF_165 _(R&D Systems; 0, 1, 10 or 50 ng/ml) for 24 h. For co-stimulation of RA SFs with TNF-α and VEGF_165_, the cells were first starved with serum-free DMEM containing 0.2% lactalbumin hydrolysate for 24 h, treated with TNF-α (1 or 10 ng/ml) for 24 h, and then stimulated with VEGF_165 _(40 ng/ml) for 24 h. HUVECs were also stimulated with these cytokines or growth factors for 24 h after being starved with serum-free medium EBM-2 containing 1% bovine serum albumin for 24 h.

To block the signaling of VEGF_165_, RA SFs that had been stimulated with TNF-α (10 ng/ml) for 24 h were incubated with SU1498 (1 or 10 μM; Calbiochem, San Diego, CA, USA), a selective VEGFR-2 tyrosine kinase inhibitor [26,27], for 30 minutes and then treated with VEGF_165 _(40 ng/ml) for 24 h. HUVECs were also treated with SU1498 in a similar way except for no treatment with TNF-α. To exclude the possible involvement of VEGFR-1 in ADAM15 expression, RA SFs and HUVECs were stimulated with recombinant human placenta growth factor (PlGF; 1, 10 or 50 ng/ml; R&D Systems), which selectively binds to VEGFR-1 [28].

### Statistics

Comparisons between two independent groups were determined by Mann-Whitney U test. Spearman's rank correlation was used for analysis of the relationship between relative ADAM15 mRNA expression and vascular density. *P*-values less than 0.05 were considered significant.

## Results

### Screening of mRNA expression of ADAMs and relative expression levels of ADAM15 in RA and OA synovial tissues

**Figure 2:**
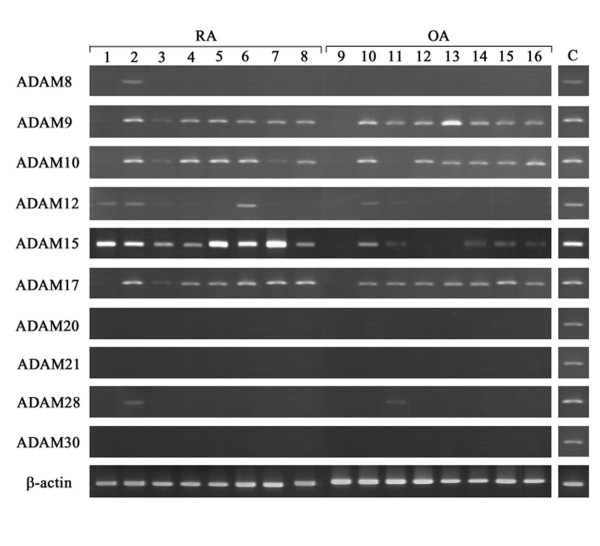
mRNA expression of ADAM species in rheumatoid arthritis (RA) and osteoarthritis (OA) synovial samples. Total RNA was extracted from eight RA (lanes 1-8) or eight OA (lanes 9-16) synovial samples, and reverse-transcribed into cDNA, followed by PCR as described in Materials and methods. C, positive controls.

**Figure 3:**
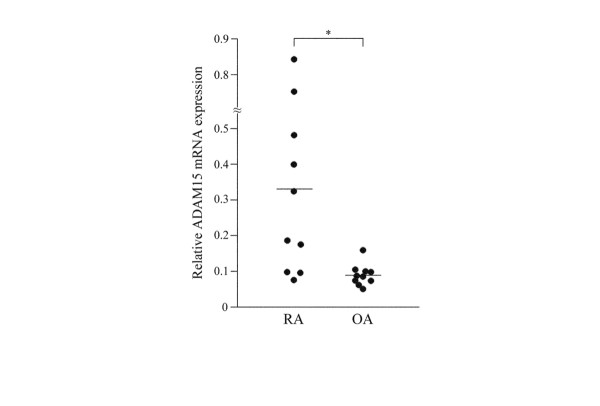
The mRNA expression levels of ADAM15 in rheumatoid arthritis (RA) or osteoarthritis (OA) synovial samples. The relative expression levels (ADAM15:ribosomal 18S ratios) were determined by real-time PCR analysis as described in Materials and methods. Bars indicate the mean values of the 10 samples of RA and OA synovial tissues. Asterisk indicates p < 0.01.

The mRNA expression of 10 different ADAMs (8, 9, 10, 12, 15, 17, 20, 21, 28 and 30) with a putative metalloproteinase motif was screened by RT-PCR analysis in eight RA and eight OA synovial samples. ADAM9, ADAM10 and ADAM17 were expressed in more than 88% of RA samples, but their expression was also observed in more than 75% of OA samples (Fig. 2). ADAM12 was expressed in 38% and 13% of RA and OA samples, respectively. ADAMs 8, 20, 21, 28 and 30 were expressed in less than 13% of both RA and OA samples. On the other hand, ADAM15 was intensely expressed in all the RA synovial samples, whereas it was detected in 63% of OA samples (Fig. 2). Because of the more selective expression of ADAM15 in RA than in OA, we focused on ADAM15 for further studies. When the expression was examined in a larger number of RA and OA samples, ADAM15 was detected in 100% of the RA samples (16 of 16 cases) and in 60% of the OA samples (12 of 20 cases) (data not shown). By real-time quantitative PCR analysis, the expression levels (ratio of ADAM15 to ribosomal 18S RNA) were significantly higher in RA samples (0.344 ± 0.276; n = 10) than in OA samples (0.091 ± 0.030; n = 10) (p < 0.01; Fig. 3).

### Expression of ADAM15 in RA synovial tissues studied by *in situ *hybridization, immunohistochemistry and immunoblotting

**Figure 4:**
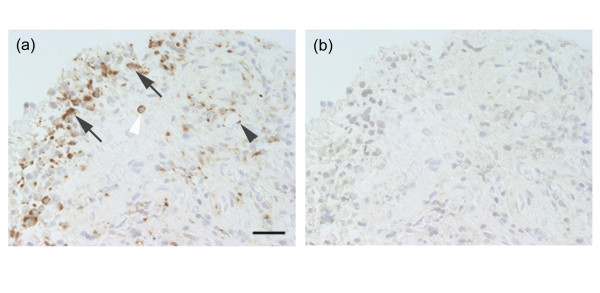
*In situ *hybridization of ADAM15 in rheumatoid arthritis synovial tissues. Paraffin sections were reacted with digoxigenin-labeled anti-sense or sense RNA probes as described in Materials and methods. Note **(a) **a positive signal in the synovial lining cells (arrows), endothelial cells (black arrowhead) and macrophage-like cells (white arrowhead) with anti-sense probe, whereas **(b) **there was only a background signal with the sense probe. Scale bar, 50 μm.

**Figure 5:**
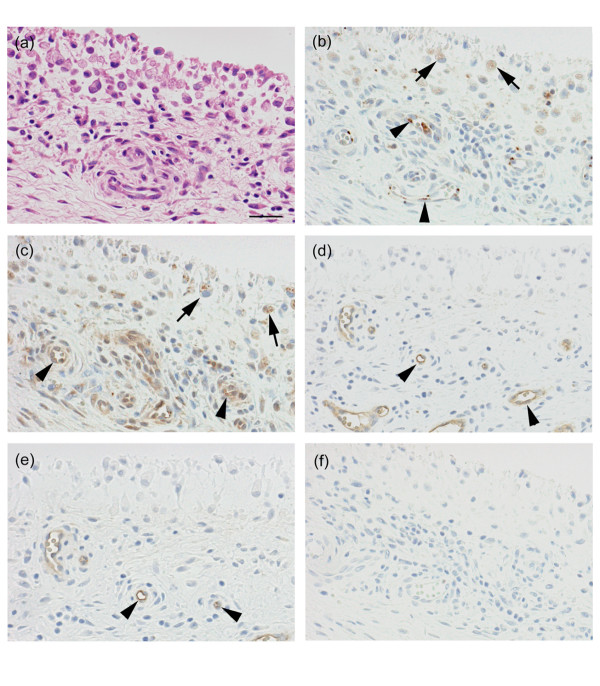
Immunolocalization of ADAM15, VEGFR-2, vWF and CD31 in rheumatoid arthritis (RA) synovial tissues. Paraffin sections were stained with **(a) **hematoxylin and eosin or immunostained with antibodies against **(b) **ADAM15, **(c) **VEGFR-2, **(d) **vWF or **(e) **CD31, or **(f) **non-immune mouse IgG as described in Materials and methods. **(b) **Note that ADAM15 is expressed in synovial lining cells and endothelial cells of blood vessels in the sublining layer. Immunostained sections were counterstained with hematoxylin. Arrows, synovial lining cells; arrowheads, endothelial cells of blood vessel. Scale bar, 100 μm.

Cells expressing ADAM15 mRNA were examined by *in situ *hybridization. Synovial lining cells, endothelial cells of blood vessels and macrophage-like cells in the sublining layer were labeled with the anti-sense RNA probe (Fig. 4a), whereas the sense probe gave only a background signal in these cells (Fig. 4b). Immunohistochemical studies showed that ADAM15 was localized to synovial lining cells, endothelial cells of blood vessels and macrophage-like cells in the sublining layer of the RA synovium (Fig. 5a,b), confirming the findings from *in situ *hybridization. No staining was obtained with non-immune IgG (Fig. 5f). VEGFR-2 was immunolocalized to some synovial lining cells and endothelial cells of blood vessels in RA samples (Fig. 5c), but vWF and CD31 were almost exclusively localized to endothelial cells (Fig. 5d,e).

**Figure 6:**
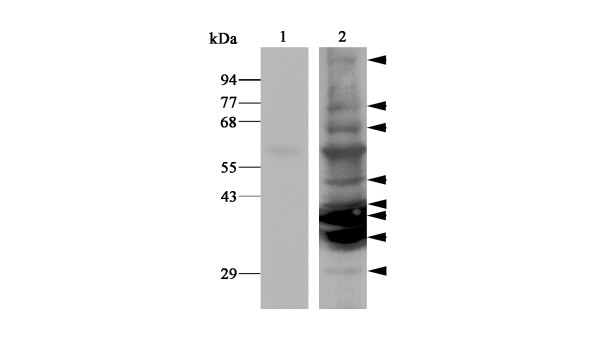
Immunoblotting of ADAM15 in rheumatoid arthritis (RA) synovial tissues. Homogenates of RA synovial tissues were prepared and subjected to immunoblotting with anti-ADAM15 antibody specific to the cysteine-rich domain of ADAM15 (240-2C7) (lane 2) or non-immune mouse IgG (lane 1) as described in Materials and methods. Immunoreactive bands of 100, 76, 66, 47, 41, 38, 34 and 29 kDa are indicated (arrow heads). The 58 kDa protein band is considered to be a non-specific band because it is also detected with non-immune IgG (lanes 1 and 2).

When homogenates of RA synovial tissues were subjected to immunoblotting analysis, eight major immunoreactive bands of 100, 76, 66, 47, 41, 38, 34 and 29 kDa were observed (Fig. 6, lane 2). Because the molecular weight of the 100-kDa band is similar to that of the precursor form of ADAM15 [12,25], this band appears to correspond to proADAM15. On the other hand, at least some of the other bands are considered to be active ADAM15 forms containing the metalloproteinase domain because of their positive immunoreactivity to the antibody specific to the cysteine-rich domain and their molecular weights. An immunoreactive band of 58 kDa was a non-specific reaction, because it was also detected with non-immune mouse IgG (Fig. 6, lane 1).

### Correlation between ADAM15 expression and vascular density in RA and OA synovial tissues

**Figure 7:**
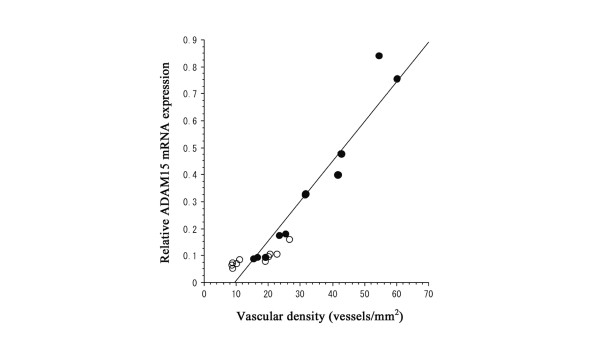
Correlation between ADAM15 mRNA expression levels and vascular density in synovial tissues. Vascular density was determined by the morphometric analysis of the CD31-immunostained sections and correlation was examined by Spearman's rank correlation. Note a direct correlation between the parameters (r = 0.907, p < 0.001; n = 20). Open and closed circles indicate osteoarthritis (OA) and rheumatoid arthritis (RA) synovial samples, respectively.

No definite correlation between relative mRNA expression levels of ADAM15 and the separate or total histological scores of RA and OA synovial tissues was observed (data not shown). Thus, we further evaluated vascular density in the RA and OA synovial tissues, by counting CD31-positive vessels in synovial tissue sections, and compared it with the mRNA expression levels of ADAM15. A linear correlation was found between expression levels and vascular density in RA and OA synovial tissues (r = 0.907, p < 0.001; n = 20; Fig. 7).

### Effect of cytokines and growth factors on ADAM15 expression in RA SFs and HUVECs

**Figure 8:**
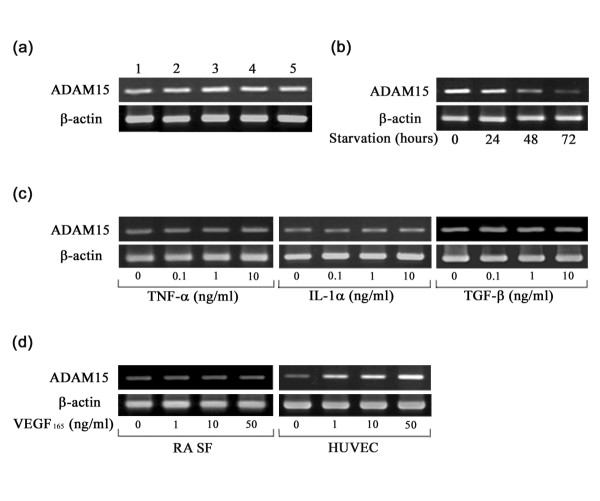
Effects of passages, starvation, cytokines and growth factors on ADAM15 expression in cultured cells. The mRNA expression of ADAM15 was examined by RT-PCR at 25 cycles as described in Materials and methods. **(a) **Effect of passages on the mRNA expression of ADAM15 in rheumatoid arthritis (RA) synovial fibroblasts (SFs). Lanes 1 to 5 indicate passages 5, 6, 7, 8 and 9 of RA SFs. **(b) **Effect of starvation on the mRNA expression of ADAM15 in RA SFs. **(c) **Effect of tumor necrosis factor (TNF)-α (0, 0.1, 1 and 10 ng/ml), IL-1α (0, 0.1, 1 and 10 ng/ml) or transforming growth factor (TGF)-β (0, 0.1, 1 and 10 ng/ml) on the mRNA expression of ADAM15 in RA SFs after stimulation with these factors for 24 h. **(d) **Regulation of the mRNA expression of ADAM15 by vascular endothelial growth factor (VEGF)_165 _in RA SFs and human umbilical vein endothelial cells (HUVECs). Cells were stimulated with VEGF_165 _(0, 1, 10 and 50 ng/ml) for 24 h. Note that VEGF_165 _enhances the expression of ADAM15 only in HUVECs.

When the expression of ADAM15 in RA SFs was examined by RT-PCR, it was constitutively expressed regardless of the passage numbers (5 to 9) of the cells (Fig. 8a). Expression was decreased to low levels in a time-dependent manner, however, after starvation with serum-free medium for up to 72 h (Fig. 8b). To test the effect of cytokines and growth factors on ADAM15 expression, RA SFs were stimulated with TNF-α, IL-1α, TGF-β or VEGF_165_; however, no changes in mRNA expression were found with these factors (Fig. 8c,d). In contrast, VEGF_165 _appeared to selectively enhance ADAM15 expression in HUVECs (Fig. 8d), whereas TNF-α, IL-1α or TGF-β did not alter the expression (data not shown). Using real-time quantitative PCR, we found that the relative expression levels of ADAM15 mRNA (ratio of ADAM15 to ribosomal 18S) in HUVECs are significantly 2.2-fold higher after treatment with VEGF_165 _(p < 0.05).

### Regulation of VEGFR-1, VEGFR-2 and neuropilin-1 expression by cytokines and growth factors in RA SFs and HUVECs

**Figure 9:**
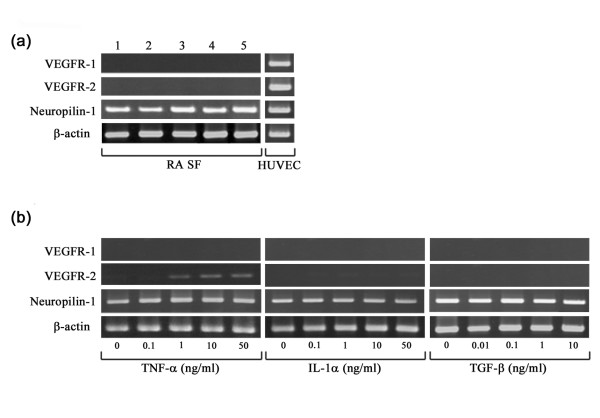
Effects of cytokines and growth factors on expression of vascular endothelial growth factor receptors (VEGFRs). The mRNA expression of the VEGFRs was examined by RT-PCR at 30 cycles as described in Materials and methods. **(a) **The expression of VEGFR-1, VEGFR-2 and neuropilin-1 in rheumatoid arthritis (RA) synovial fibroblasts (SFs) of different passages and human umbilical vein endothelial cells (HUVECs). Lanes 1 to 5 correspond to RA SFs of passages 5, 6, 7, 8 and 9, respectively. **(b) **The mRNA expression of VEGFR-1, VEGFR-2 and neuropilin-1 in RA SFs after 24 h stimulation with tumor necrosis factor (TNF)-α (0, 0.1, 1, 10 and 50 ng/ml), IL-1α (0, 0.1, 1, 10 and 50 ng/ml) or transforming growth factor (TGF)-β (0, 0.01, 0.1, 1 and 10 ng/ml). Note that TNF-α selectively induces the mRNA expression of VEGFR-2 in RA SFs.

As previously reported [15,29], HUVECs expressed the three major VEGF receptors (VEGFR-1, VEGFR-2 and neuropilin-1), but RA SFs expressed only neuropilin-1 under unstimulated conditions (Fig. 9a). Because the data that VEGF_165 _stimulated ADAM15 expression only in HUVECs suggested that the effect is dependent on the expression of VEGF receptors, we tried to induce VEGF receptors by treating RA SFs with cytokines and growth factor and found that TNF-α, but not IL-1α or TGF-β, can induce VEGFR-2 expression without affecting the expression of VEGFR-1 or neuropilin-1 (Fig. 9b).

### Stimulation of ADAM15 expression by VEGF_165 _in VEGFR-2-expressing RA SFs

**Figure 10:**
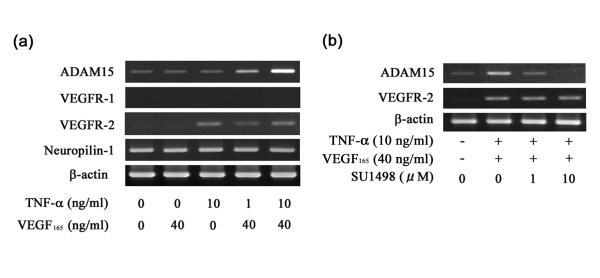
Effects of tumor necrosis factor-α, vascular endothelial growth factor_165 _and SU1498 on ADAM15 expression. **(a) **Effect of tumor necrosis factor (TNF)-α and vascular endothelial growth factor (VEGF)_165 _on ADAM15 expression. After starvation, rheumatoid arthritis (RA) synovial fibroblasts (SFs) were treated with 10 ng/ml TNF-α and/or 40 ng/ml VEGF_165 _for 24 h, and then the expression of ADAM15, vascular endothelial growth factor receptor (VEGFR)-1, VEGFR-2 and neuropilin-1 was examined by RT-PCR at 25 cycles (ADAM15 and β-actin) or 30 cycles (VEGFR-1, VEGFR-2 and neuropilin-1) as described in Materials and methods. Note that treatment with TNF-α induces VEGFR-2 and co-treatment with TNF-α and VEGF_165 _stimulates the mRNA expression of ADAM15 in RA SFs. **(b) **Effect of SU1498 on ADAM15 expression. After starvation for 24 h, RA SFs were stimulated with 10 ng/ml TNF-α for 24 h to induce VEGFR-2. Then, the cells were treated with SU1498 (0, 1 and 10 μM) for 30 minutes and stimulated with 40 ng/ml VEGF_165 _for 24 h. The mRNA expression of ADAM15 was determined by RT-PCR at 25 cycles as described in Materials and methods. Note that the stimulated expression of ADAM15 mRNA is inhibited by the treatment with SU1498, while the expression of VEGFR-2 mRNA is not affected by the treatment.

As TNF-α induced VEGFR-2 expression in RA SFs, we further examined whether VEGF_165 _enhances ADAM15 expression in VEGFR-2-expressing RA SFs. After stimulation of RA SFs with TNF-α or VEGF_165 _alone, ADAM15 mRNA expression, which was only weak after starvation of the cells, did not change (Fig. 10a). When the cells were sequentially treated with TNF-α and VEGF_165_, however, the level of ADAM15 expression appeared to be increased (Fig. 10a). Real-time quantitative PCR analysis demonstrated that the expression levels are significantly 2.2-fold higher in RA SFs treated with TNF-α and VEGF_165 _compared with the control without treatment (p < 0.05).

### VEGFR-2 signaling in VEGF_165_-stimulated ADAM15 expression in RA SFs

To examine the involvement of VEGFR-2 signaling in the stimulation of ADAM15 expression with TNF-α and VEGF_165_, RA SFs were incubated with SU1498, a selective VEGFR-2 tyrosine kinase inhibitor, prior to the stimulation with VEGF_165 _and ADAM15 mRNA expression was examined. ADAM15 expression decreased with 1 μM SU1498 and was completely suppressed with 10 μM SU1498, while VEGFR-2 expression was not affected by the treatment with such concentrations of the inhibitor (Fig. 10b). The enhanced expression of ADAM15 in HUVECs was also inhibited by the treatment with SU1498 (data not shown). PlGF, which selectively binds to VEGFR-1, did not affect the mRNA expression of ADAM15 in either RA SFs or HUVECs (data not shown).

### Immunohistochemical demonstration of ADAM15 expression and VEGFR-2 induction by TNF-α in RA SFs

**Figure 11:**
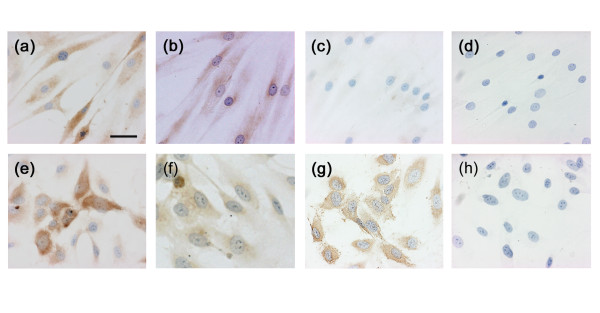
Immunohistochemistry of ADAM15, vascular endothelial growth factor receptor (VEGFR)-2 and von Willebrand factor (vWF). **(a-d) **Rheumatoid arthritis (RA) synovial fibroblasts (SFs) and **(e-h) **human umbilical vein endothelial cells (HUVEC) were cultured on Lab-Tek II chamber slides and immunostained with antibodies against **(a,e) **ADAM15, **(b,f) **VEGFR-2 or **(c,g) **vWF or **(d,h) **non-immune mouse IgG as described in Materials and methods. Immunostaining of VEGFR-2 in RA SFs **(b) **was performed with RA SFs that were treated with 10 ng/ml TNF-α for 24 h prior to immunohistochemistry. Note that vWF is not immunostained in RA SFs **(c)**, but VEGFR-2 is expressed in those stimulated with TNF-α **(b)**. Scale bar, 25 μm.

Protein expression of ADAM15 and endothelial cell markers in cultured RA SFs was examined by immunohistochemistry. ADAM15 was immunolocalized to RA SFs and HUVECs (Fig. 11a,e), whereas no staining was observed with non-immune IgG (Fig. 11d,h). On the other hand, although VEGFR-2, vWF and CD31 were all immunostained in HUVECs (VEGFR-2 and vWF, Fig. 11f,g; CD31, data not shown), RA SFs were negative for these endothelial cell markers (vWF, Fig. 11c; VEGFR-2 and CD31, data not shown). When RA SFs were treated with TNF-α for 24 h, however, they were positively immunostained with anti-VEGFR-2 antibody (Fig. 11b). In accordance with the immunohistochemical data, the mRNA expression of CD31 and vWF in untreated RA SFs was not detected by RT-PCR (data not shown).

## Discussion

In the present study, we have demonstrated that among the 10 different ADAM species with the putative metalloproteinase motif, ADAM15 is more frequently and intensely expressed in RA synovium than in OA synovium. The mRNA expression patterns of the ADAM species in synovial tissues could be classified into three groups: constitutive expression in both RA and OA samples (ADAM9, ADAM10 and ADAM17); negligible or no expression in RA or OA (ADAM8, ADAM20, ADAM21, ADAM28 and ADAM30); and more selective expression in RA than in OA (ADAM12 and ADAM15). When the expression patterns were compared with those in human astrocytic tumor and normal brain tissues [9], they were different in that more ADAM species, including ADAM9, ADAM10, ADAM15, ADAM17, ADAM20, ADAM21 and ADAM28, are constitutively expressed in brain tumor and normal brain tissues, but similar in that the expression of ADAM8 and ADAM30 is negligible. ADAM12 was selectively overexpressed in the highly malignant glioblastomas and appeared to play a key role in the tumor cell proliferation through shedding of heparin-binding epidermal growth factor [9]. This was not the case in RA synovium, however, because ADAM12 expression was confined to less than 40% of the RA samples and the expression level did not correlate with synovial lining cell hyperplasia.

A study by Bohm and co-workers [12] described the expression of ADAM15 in RA and OA synovial tissues by immunohistochemistry and *in situ *hybridization, but their study did not quantitatively analyze the expression levels. The present study has provided the first evidence that the mRNA expression level of ADAM15 is significantly 3.8-fold higher in RA than in OA. Our data of *in situ *hybridization and immunohistochemistry in RA synovium demonstrated that synovial lining cells, endothelial cells of blood vessels and macrophage-like cells in the sublining layer are responsible for the expression of ADAM15. The finding confirms the previous observation that synovial lining cells and macrophage-like cells express ADAM15 [12], but further indicate that endothelial cells, which are positive for CD31 and vWF, express ADAM15 in RA synovium. The expression by RA synovial lining cells and endothelial cells was also supported by our immunohistochemical data with cultured RA SFs and HUVECs. Interestingly, several ADAM15 species with molecular weights ranging from 100 kDa to 29 kDa were immunoblotted with RA synovial tissues. The data suggest that proADAM15 is susceptible to proteolytic cleavages and processed into fragments including active forms in RA synovial tissues.

One of the important findings in the present study is that VEGF_165 _up-regulates the expression of ADAM15. Overexpression of ADAM species is known in tumor cell lines, for example, ADAM10, ADAM12 and ADAM15 in hematological malignant tumor cell lines [30], and ADAM9, ADAM10, ADAM15 and ADAM17 in prostate cancer cell lines [31]. Although dihydrotestosterone modulates the gene expression of ADAM9, ADAM10 and ADAM17 in an androgen-dependent cell line [32], the expression of the ADAM species in these tumor cell lines may result mainly from the gene regulation associated with transformation of the cells. Phorbol 12-myristate 13-acetate stimulates human monocyte-like cell line THP-1 to enhance expression of ADAM8, ADAM9 and ADAM17, but decreases ADAM15 expression [33]. In addition, platelet-derived growth factor stimulates human vascular smooth muscle cells to overexpress ADAM9 and ADAM15 [34]. In human OA chondrocytes, IL-1 and/or retinoic acid up-regulate ADAM15 and ADAM17, but down-regulate ADAM9 [35]. In the present study, however, we have shown that VEGF_165_, but not IL-1α, TNF-α or TGF-β, stimulates HUVECs to enhance the gene expression of ADAM15. In addition, our data indicate that TNF-α induces VEGFR-2 expression in RA SFs, and VEGF_165 _up-regulates ADAM15 expression in the TNF-α-stimulated RA SFs. The specific involvement of VEGFR-2 signaling in the enhanced ADAM15 gene expression was demonstrated by the findings that the stimulation was blocked with SU1498, an inhibitor of VEGFR-2, and that PlGF, which selectively binds to VEGFR-1, had no effect on ADAM15 expression. Thus, these results demonstrate for the first time that VEGF_165 _is a stimulator of ADAM15 gene expression in cells that express VEGFR-2. Although VEGFR-2 expression was originally thought to be specific to endothelial cells, accumulated evidence indicates that many cell types, such as hematopoietic stem cells [36], megakaryocytes [36], retinal progenitor cells [37] and OA chondrocytes [19], express the receptor. Because RA synovial fluids contain high amounts of TNF-α and VEGF_165 _[38,39], it is reasonable to think that synovial lining cells are co-stimulated with these factors to induce VEGFR-2 and ADAM15 in RA synovial tissue. In fact, VEGFR-2 and ADAM15 were immunolocalized to RA synovial lining cells in the present study, suggesting the validity of the hypothesis.

The present study has demonstrated that the expression levels of ADAM15 directly correlate with the vascular density of synovial tissues. This suggests the possible involvement of ADAM15 in the angiogenic steps of RA synovial tissues, which include sprouting, migration and proliferation of endothelial cells, and maturation of vessels [40]. The notion that ADAM15 may be implicated in pathological angiogenesis is supported by recent experimental data: ADAM15 is up-regulated on the angiogenic endothelial cell surface [41]; ADAM15 has proteinase activity to digest gelatin and type IV collagen [42], which is essential for endothelial cells to sprout and migrate; ADAM15 interacts with αvβ3 and α5β1 integrins [43], which promote cell migration and proliferation of endothelial cells and smooth muscle cells [44,45]; inhibition of ADAM15's functions by antibodies, antisense oligonucleotide and metalloproteinase inhibitor decreases cell migration [42]; ADAM15-deficient mice show reduced neovascularization in hypoxia-induced proliferative retinopathy compared to wild-type control mice [8]; and the recombinant human disintegrin domain of ADAM15 itself exhibits anti-angiogenic activity, probably by disturbing the interaction between ADAM15 and integrins, leading to inhibition of endothelial cell migration and proliferation [46]. In addition, Ham *et al*. [47] have reported that ADAM15 is co-localized with vascular endothelial cadherin in the adherens junctions of endothelial cells and this cadherin drives ADAM15 to the cell junctions. Moreover, overexpression of ADAM15 is known to enhance cell-cell interactions [48]. VEGF is highly expressed in RA synovial tissues and believed to play a central role in synovial angiogenesis [49], and it stimulates ADAM15 expression in endothelial cells as shown in the present study. Altogether, these data suggest the possibility that ADAM15 may play a role in maturation of the blood vessels newly formed by VEGF-induced angiogenesis through enhancing endothelial cell-cell interaction in the RA synovium.

The biological significance of the overexpression of ADAM15 in RA synovial lining cells and macrophage-like cells is not clear at present. The fact that RA synovial lining cells express α5β1 and αvβ3 integrins [50-52], however, suggests the possibility that ADAM15 may function as a binding molecule to reinforce cell-cell and/or cell-ECM interactions. On the other hand, because ADAM15 is reported to have proteolytic activities towards ECM components (gelatin and type IV collagen) [42], it is possible that ADAM15 may be involved in the degradation of cartilage ECM at the synovial cartilage junction. In addition, ADAM15 is known to produce soluble forms of CD23 through ectodomain shedding of membrane-bound CD23 on cell membranes of inflammatory cells [53]. Soluble forms of CD23, which are elevated in synovial fluid and sera of RA patients [54-56], promote the production of inflammatory cytokines by macrophages [57] and stimulate monocytes and T cells [58]. Thus, these data suggest that ADAM15 may play a role in the aggravation of inflammation of RA synovitis through production of soluble forms of CD23, which trigger inflammatory process via monokine release and stimulate monocytes and T cells [59,60]. In contrast to the prospective role of ADAM15 in joint destruction, a recent study has demonstrated that aging mice with a targeted disruption of ADAM15 exhibit accelerated development of OA changes in the articular cartilage, and suggested that ADAM15 expressed by chondrocytes has a homeostatic, rather than a destructive, role in the cartilage [61]. Thus, further studies are definitely needed to elucidate the exact role of this unique and multifunctional molecule in RA and OA.

## Conclusion

Our results demonstrate that ADAM15 is overexpressed in lining cells, endothelial cells of blood vessels and macrophage-like cells in the sublining layer of RA synovium, with a direct correlation with vascular density in the synovium, and that the expression of ADAM15 in RA SFs is up-regulated by the action of VEGF_165 _via VEGFR-2. These data suggest that ADAM15 is involved in angiogenesis in the RA synovium.

## Abbreviations

ADAM = a disintegrin and metalloproteinase; DMEM = Dulbecco's modified Eagle's medium; ECM = extracellular matrix; HUVEC = human umbilical vein endothelial cells; IL = interleukin; MMP = matrix metalloproteinase; OA = osteoarthritis; PlGF = placenta growth factor; RA = rheumatoid arthritis; RT-PCR = reverse transcription polymerase chain reaction; SF = synovial fibroblast; TGF = transforming growth factor; TNF = tumor necrosis factor; VEGF = vascular endothelial growth factor; VEGFR = vascular endothelial growth factor receptor; vWF = von Willebrand factor.

## Competing interests

The authors declare that they have no competing interests.

## Authors' contributions

KK carried out critical examinations in this study, especially histopathological analyses, RT-PCR, real-time PCR, *in situ *hybridization, characterization of monoclonal anti-ADAM15 antibody, immunohistochemistry, immunoblotting, cell cultures, and stimulation of cells, and drafted the manuscript as a part of his doctoral thesis, with the assistance of the coauthors. HE and SO prepared histological specimens and carried out RNA extraction. II participated in the cultures and stimulation of cells. YF participated in the *in situ *hybridization. EI participated in the vascular density analyses. EO carried out the development of the monoclonal antibody against human ADAM15. YT gave critical suggestions concerning orthopedics and experimental design. HM carried out the clinical studies of each case and performed surgery to obtain synovial tissues with the written informed consent of patients. YO conceived of the study, participated in its design and coordination, and is the corresponding author. All authors read and approved the final manuscript.

## Figures and Tables

**Table 1:** Sequences of the primers used for RT-PCR

Primer's name	Product	Oligonucleotide sequence	Size (base pairs)	Accession number
ADAM8	Forward	5'-GCCGTCTTCAGGCCTCGGCCCGGGGACTCT-3'	651	NM001109
	Reverse	5'-AGGGGCGTTGGCGAGGCACACCGACTGCGG-3'		
ADAM9	Forward	5'-GCTGTCTTGCCACAGACCCGGTATGTGGAG-3'	604	HSU41766
	Reverse	5'-TGGAATATTAAGAAGGCAGTTTCCTCCTTT-3'		
ADAM10	Forward	5'-ATCCAGTCATGTTAAAGCGATTGATACAATTTAC-3'	434	NM001110
	Reverse	5'-TCCAAAGTTATGTCCAACTTCGTGAGCAAAAGTAA-3'		
ADAM12	Forward	5'-GAGACCCTCAAGGCAACTAAGTATGTGGAG-3'	627	AF023476
	Reverse	5'-CGGCAGGTTAAACAGGCACACCCCCATTCC-3'		
ADAM15	Forward	5'-CTGGGACAGCGCCACATTCGCCGGAGGCGG-3'	688	HSU41767
	Reverse	5'-TCCGCAGAAAGCAGCCATAGGGGGTAGGCT-3'		
ADAM17	Forward	5'-AGAGCTGACCCAGATCCCATGAAGAACACG-3'	777	HSU69611
	Reverse	5'-GCGTTCTTGAAAACACTCCTGGGCCTTACT-3'		
ADAM20	Forward	5'-AAAATAGCACACCAGATGGAGTTGCAATTG-3'	702	AF029899
	Reverse	5'-ATTCCCACAGTACTTCAGTCTAAATATATT-3'		
ADAM21	Forward	5'-TCTGGCTTGGGGTATTTTTG-3'	500	AF158644
	Reverse	5'-TTGGCGTGCTACTTCCTTCT-3'		
ADAM28	Forward	5'-GCTGTGATGCTAAGACATGT-3'	871	AF137334
	Reverse	5'-TGAACAGCCTTTACCATCTG-3'		
ADAM30	Forward	5'-AACCAGGTGCCAACTGTAGC-3'	496	AF171932
	Reverse	5'-CCCATGGGTTTCATGGATAG-3'		
VEGFR-1	Forward	5'-GATGTTGAGGAAGAGGAGGATT-3'	1146	NM002019
	Reverse	5'-AAGCTAGTTTCCTGGGGGTATA-3'		
VEGFR-2	Forward	5'-GATGTGGTTCTGAGTCCGTCT-3'	562	NM002253
	Reverse	5'-CATGGCTCTGCTTCTCCTTTG-3'		
Neuropilin-1	Forward	5'-CAACGATAAATGTGGCGATACT-3'	824	NM003873
	Reverse	5'-TATACTGGGAAGAAGCTGTGAT-3'		
CD31	Forward	5'-CAACGAGAAAATGTCAGA-3'	259	NM000442
	Reverse	5'-GGAGCCTTCCGTTCTAGAGT-3'		
vWF	Forward	5'-GTTCGTCCTGGAAGGATCGG-3'	697	NM000552
	Reverse	5'-CACTGACACCTGAGTGAGAC-3'		
β-Actin	Forward	5'-TGACGGGGTCACCCACACTGTGCCCATCTA-3'	661	NM001101
	Reverse	5'-CTAGAAGCATTTGCGGTGGACGATGGAGGG-3'		

ADAM, a disintegrin and metalloproteinase; VEGFR, vascular endothelial growth factor receptor; vWF, von Willebrand factor.
