# Correction: Expression of ADAM15 in rheumatoid synovium: up-regulation by vascular endothelial growth factor and possible implications for angiogenesis

**DOI:** 10.1186/s13075-022-02936-1

**Published:** 2022-11-01

**Authors:** Koichiro Komiya, Hiroyuki Enomoto, Isao Inoki, Satoko Okazaki, Yoshinari Fujita, Eiji Ikeda, Eiko Ohuchi, Hideo Matsumoto, Yoshiaki Toyama, Yasunori Okada

**Affiliations:** 1 Department of Pathology, School of Medicine, Keio University, Shinjuku-ku, Tokyo, Japan; 2 Department of Orthopaedic Surgery, School of Medicine, Keio University, Shinjuku-ku, Tokyo, Japan; 3 Biopharmaceutical Department, Daiichi Fine Chemical Co. Ltd., Takaoka, Toyama, Japan

**Correction: Arthritis Res Ther 7, R1158 (2005)**

**https://doi.org/10.1186/ar1796**

Following publication of the original article [1], an error was identified in Figs. 2 and 5. The correct figures are given below.

**Fig. 2:**
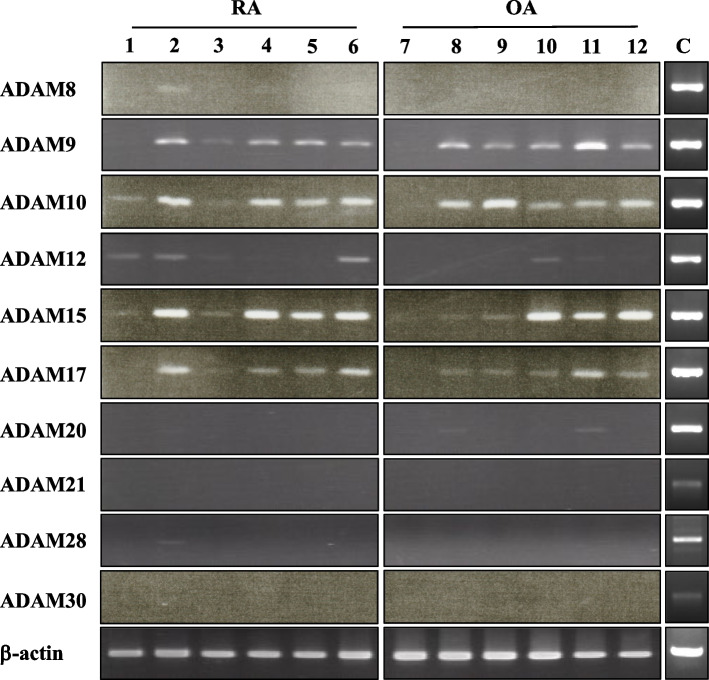
mRNA expression of ADAM species in rheumatoid arthritis (RA) and osteoarthritis (OA) synovial samples. Total RNA was extracted from RA or OA synovial tissue samples, and reverse transcribed into cDNA, followed by PCR as described in Materials and methods. Results from representative six RA (lanes 1-6) and six OA (lanes 7-12) synovial samples are shown. Although sample numbers of RA and OA cases were reduced from eight to six, the data in this corrected version of Fig. 2 are confirmatory to those in the original Fig. 2. C, positive controls. The cropped gel images in the corrected Fig. 2 are delineated, and the uncropped images for ADAM species and β–actin (full-length gels) are uploaded as ESM

**Fig. 5:**
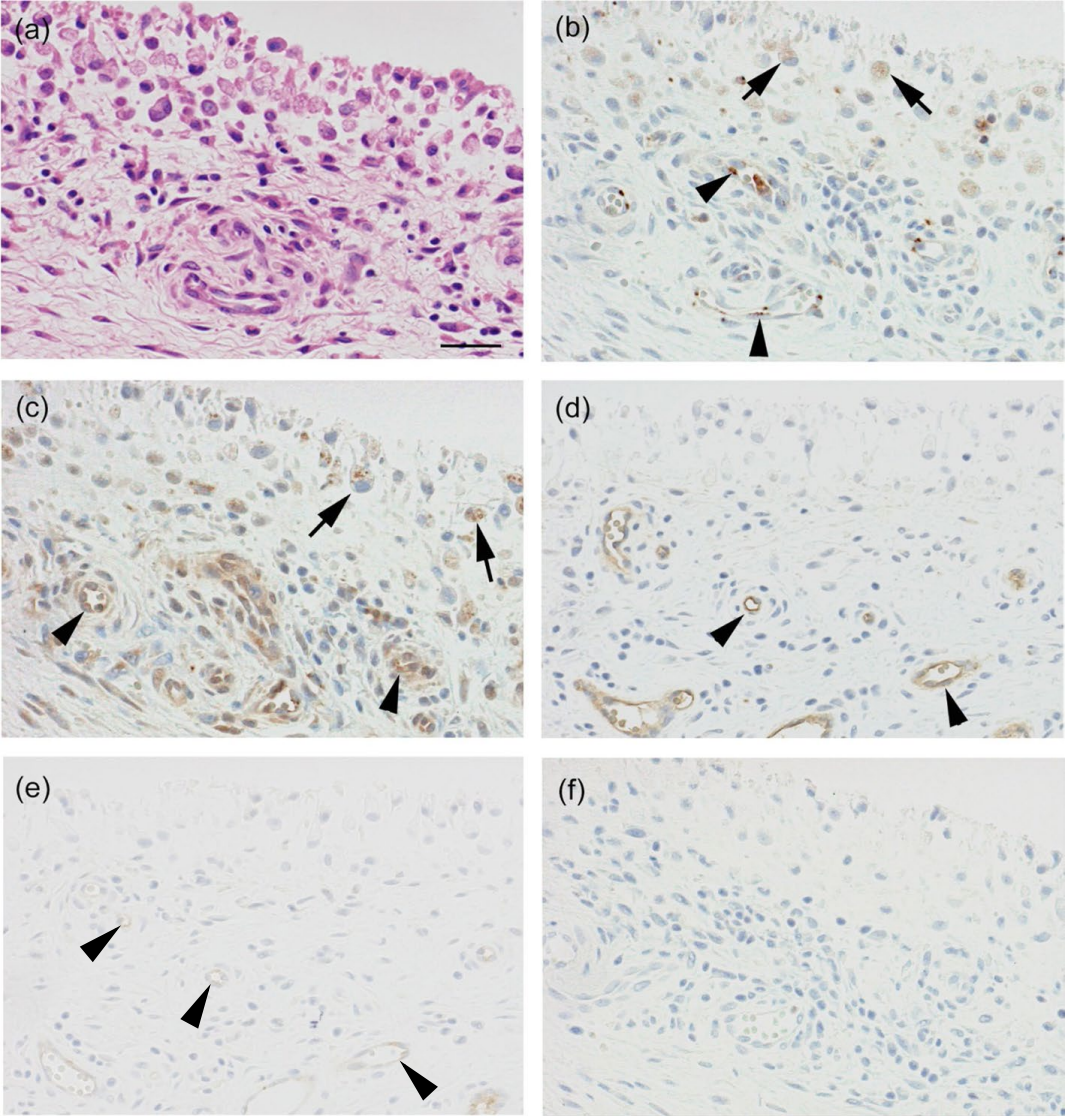
Immunolocalization of ADAM15, VEGFR-2, vWF and CD31 in rheumatoid arthritis (RA) synovial tissues. Paraffin sections were stained with **a** hematoxylin and eosin or immunostained with antibodies against **b** ADAM15, **c** VEGFR-2, **d** vWF or **e** CD31, or **f** non-immune mouse IgG as described in Materials and methods. Note that ADAM15 is expressed in synovial lining cells and endothelial cells of blood vessels in the sublining layer (**b**). Immunostained sections were counterstained with hematoxylin. Arrows, synovial lining cells; arrowheads, endothelial cells of blood vessel. Scale bar, 100 μm

In addition, since the publication of the original article [1], it has come to our attention that we failed to include information for one of the authors in the competing interests section. The full competing interests section for this article can be found below.

## Supplementary Information

**Additional file 1.** Supplementary figures.

## References

[1] Komiya K, Enomoto H, Inoki I (2005). Expression of ADAM15 in rheumatoid synovium: up-regulation by vascular endothelial growth factor and possible implications for angiogenesis. Arthritis Res Ther.

