# Supplementary material for: Correction: Expression of ADAM15 in rheumatoid synovium: up-regulation by vascular endothelial growth factor and possible implications for angiogenesis
Source: Arthritis Res Ther. 2022 Nov 1;24:244. doi: 10.1186/s13075-022-02936-1 (PMC9624011; doi:10.1186/s13075-022-02936-1)
Supplement: Supplementary file 1 — Additional file 1. Supplementary figures. [file 13075_2022_2936_MOESM1_ESM.pdf]

Full-length gels for the corrected version of Figure 2

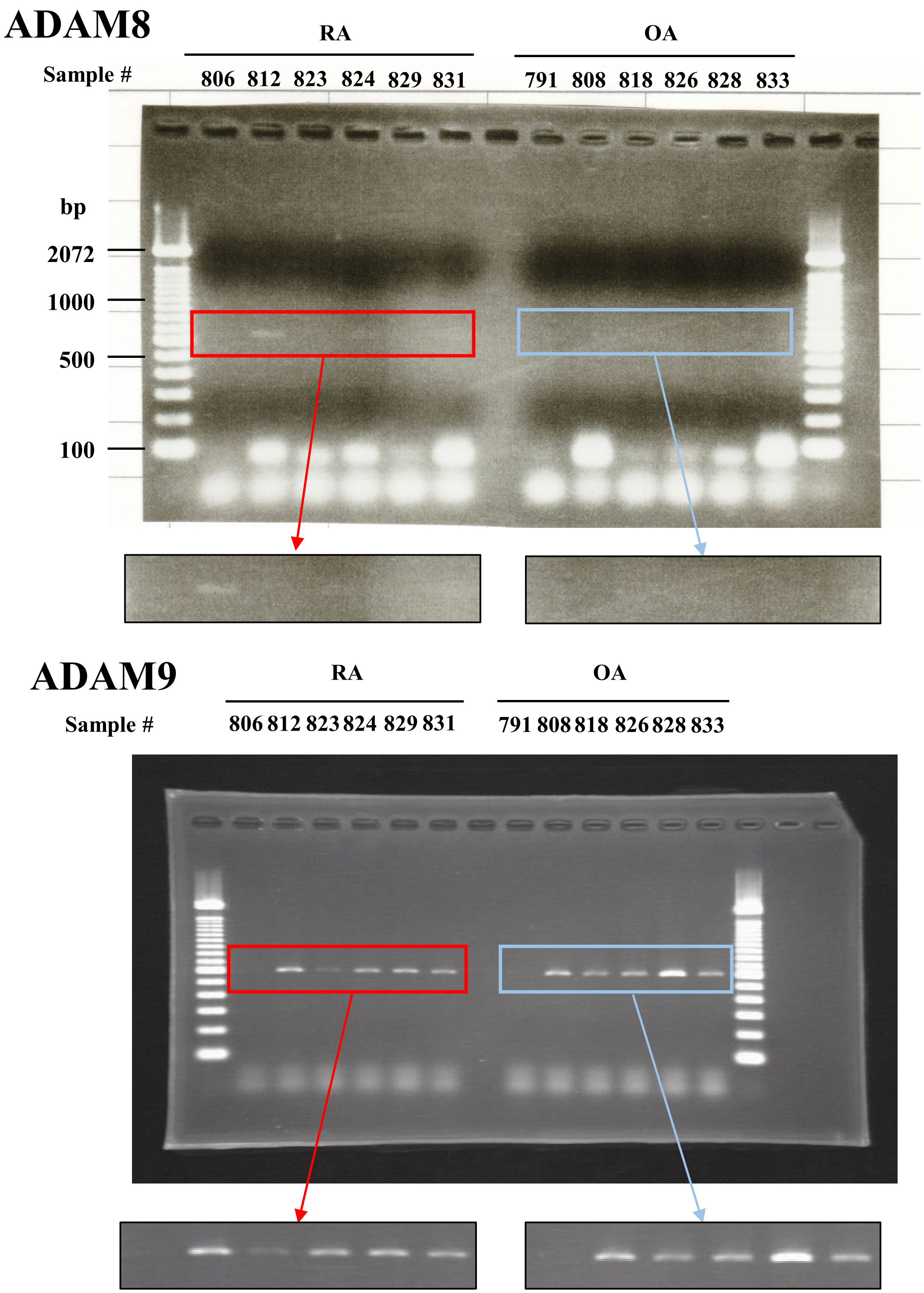

Full-length gels corresponding to ADAM8 and ADAM9 in the corrected version of Figure 2. Images of ADAM8 and ADAM9 were obtained by scanning the image mounted on the notebook and from the electronic file, respectively. Adobe Photoshop was used to crop the indicated bands and the bands were placed in Figure 2.

Full-length gels for the corrected version of Figure 2

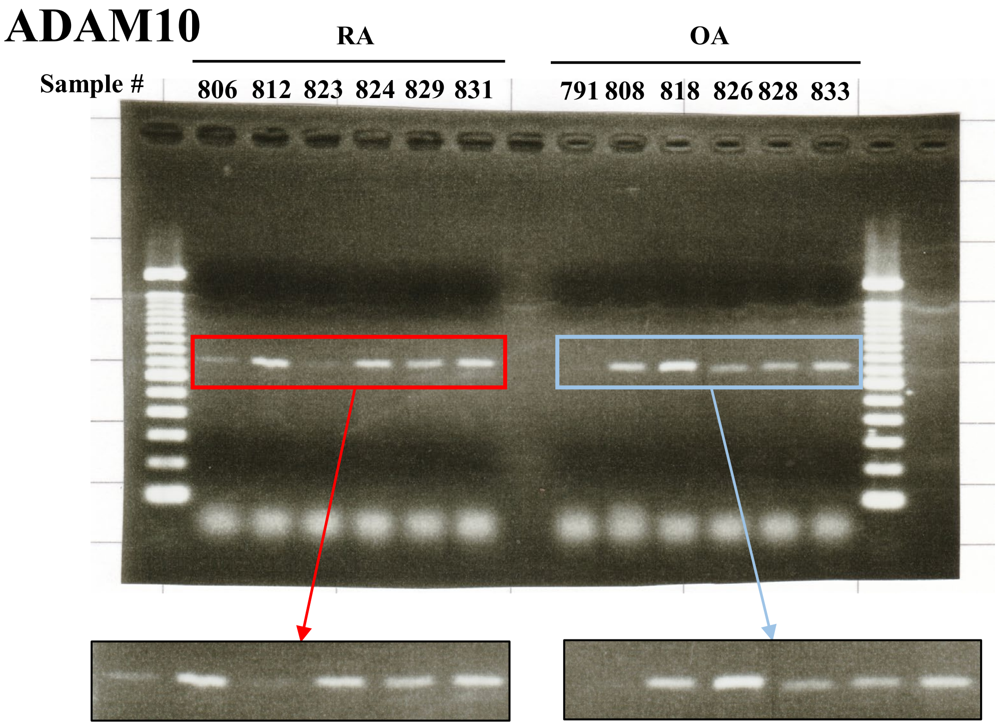

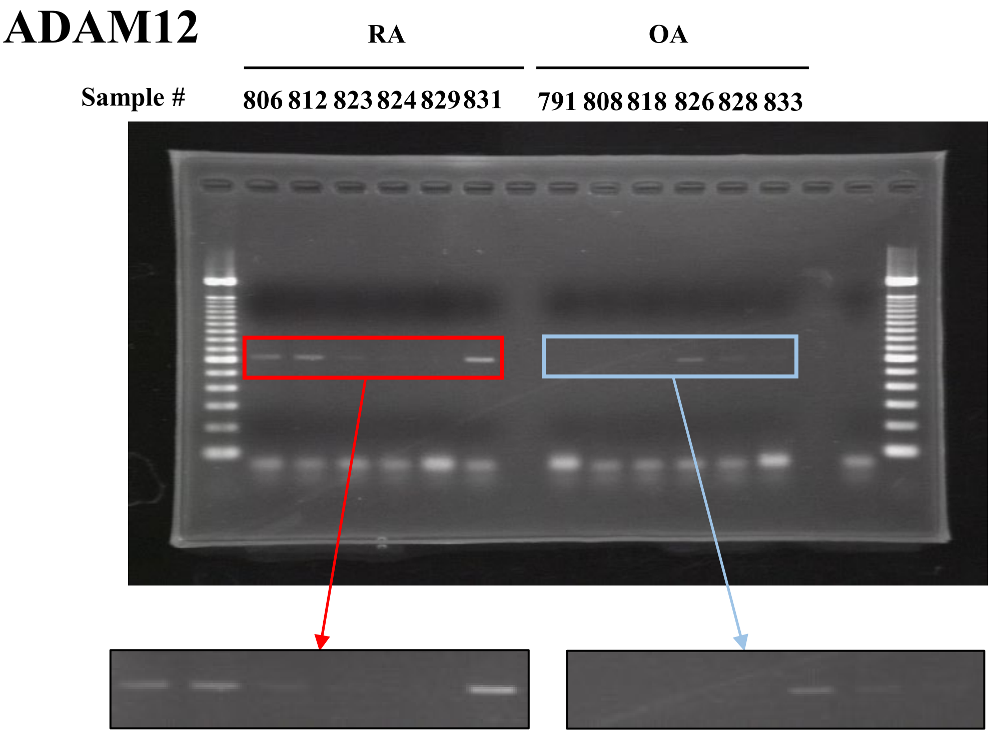

Full-length gels corresponding to ADAM10 and ADAM12 in the corrected version of Figure 2. Images of ADAM10 and ADAM12 were obtained by scanning the image mounted on the notebook and from the electronic file, respectively. Adobe Photoshop was used to crop the indicated bands and the bands were placed in Figure 2.

Full-length gels for the corrected version of Figure 2

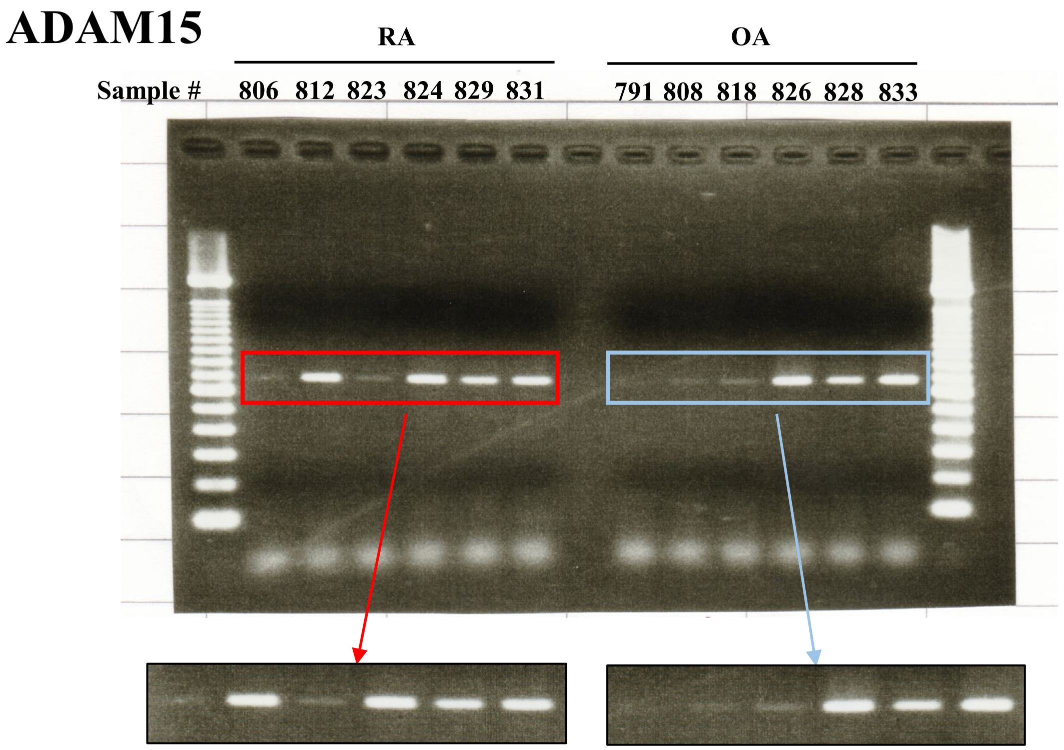

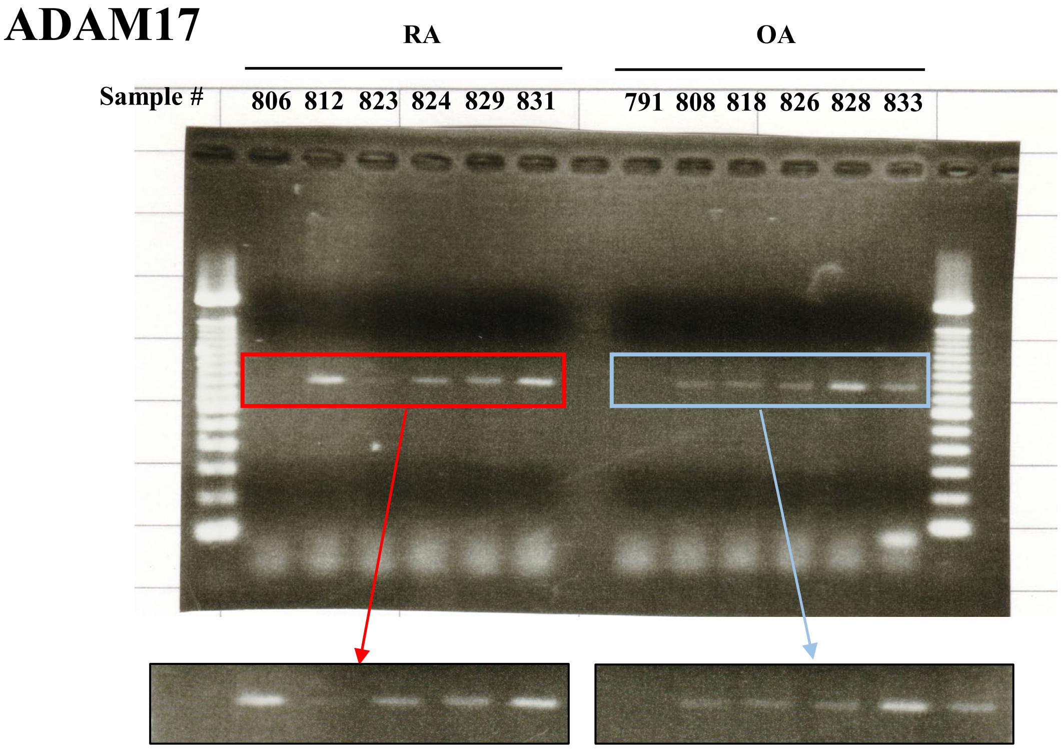

Full-length gels corresponding to ADAM15 and ADAM17 in the corrected version of Figure 2. Images of ADAM15 and ADAM17 were obtained by scanning the images mounted on the notebook. Adobe Photoshop was used to crop the indicated bands and the bands were placed in Figure 2.

Full-length gels for the corrected version of Figure 2

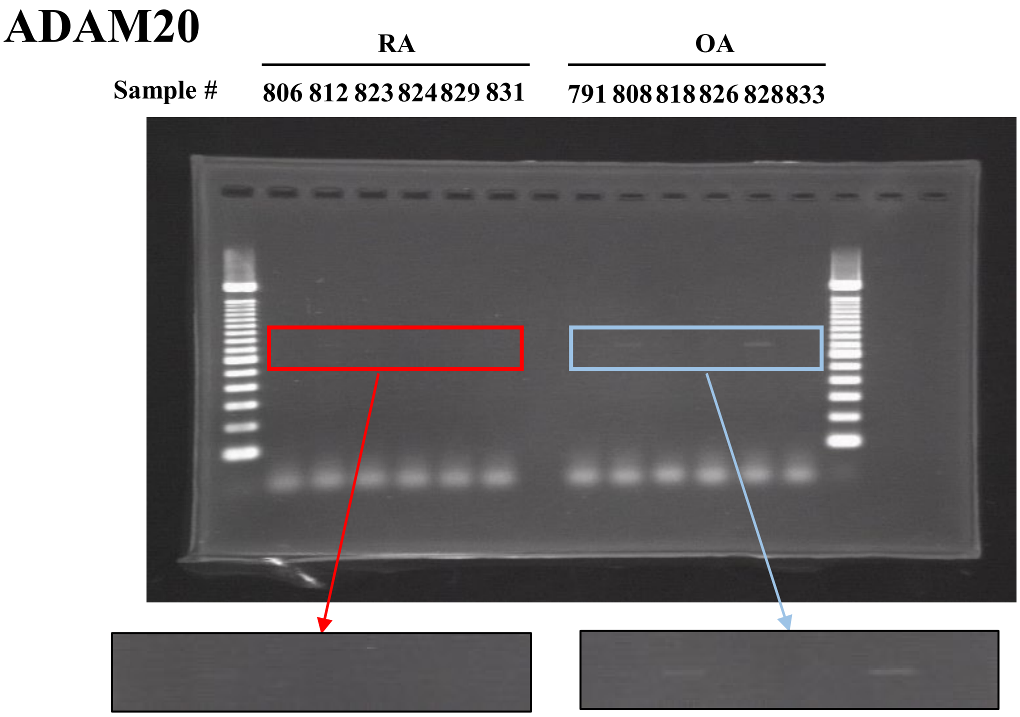

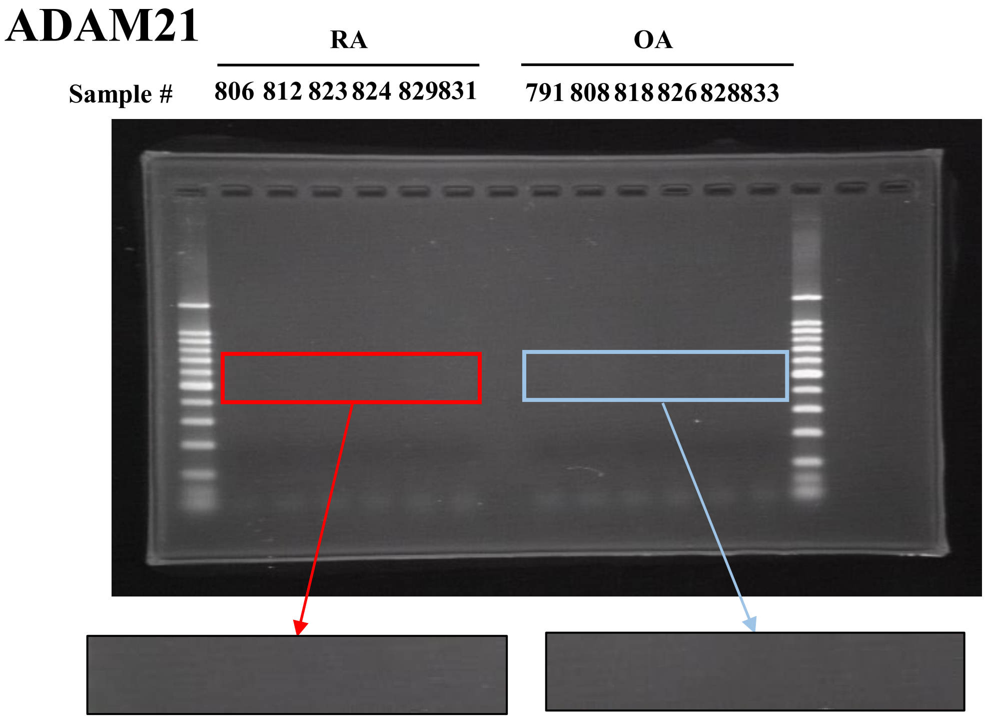

Full-length gels corresponding to ADAM20 and ADAM21 in the corrected version of Figure 2. Images of ADAM20 and ADAM21 were obtained from the electronic files. Adobe Photoshop was used to crop the indicated band areas and the areas were placed in Figure 2.

Full-length gels for the corrected version of Figure 2

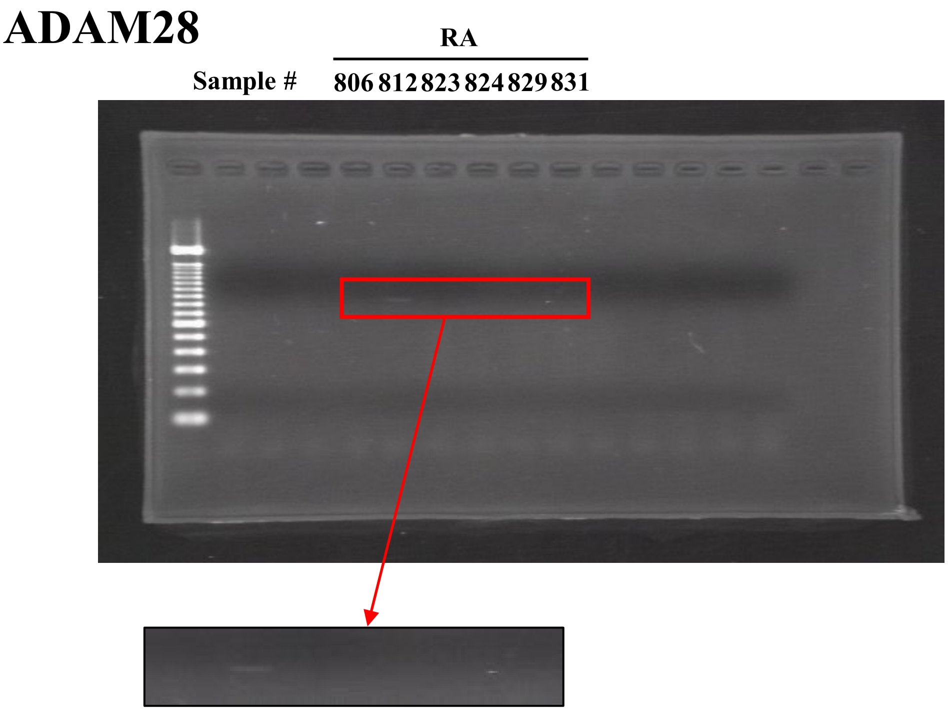

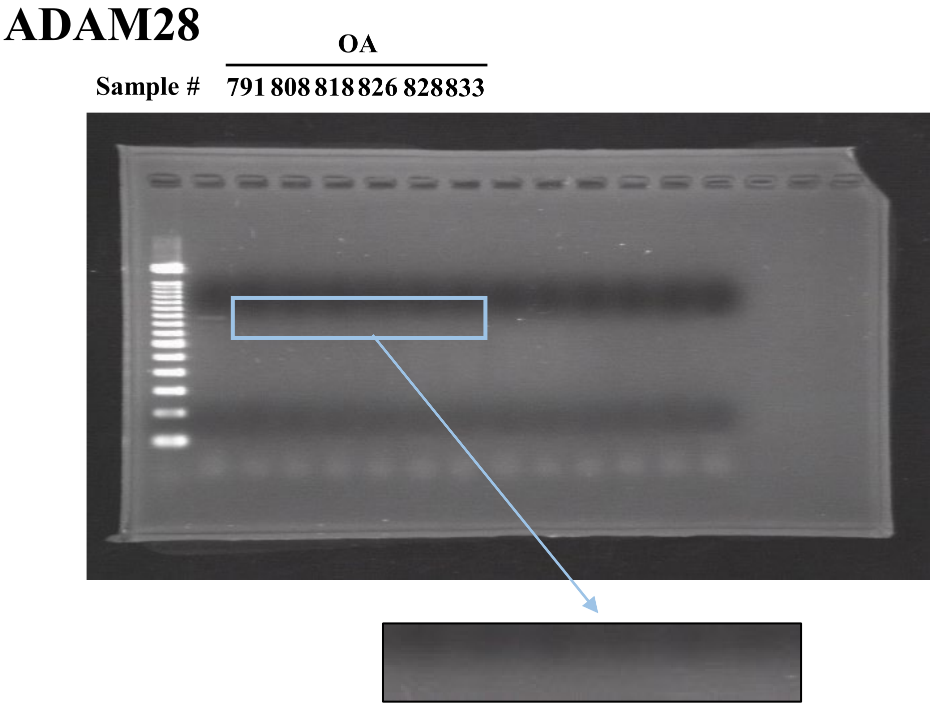

Full-length gels corresponding to ADAM28 in the corrected version of Figure 2. Images of ADAM28 in the RA and OA samples were obtained from the electronic files. Adobe Photoshop was used to crop the indicated band areas and the areas were placed in Figure 2.

Full-length gels for the corrected version of Figure 2

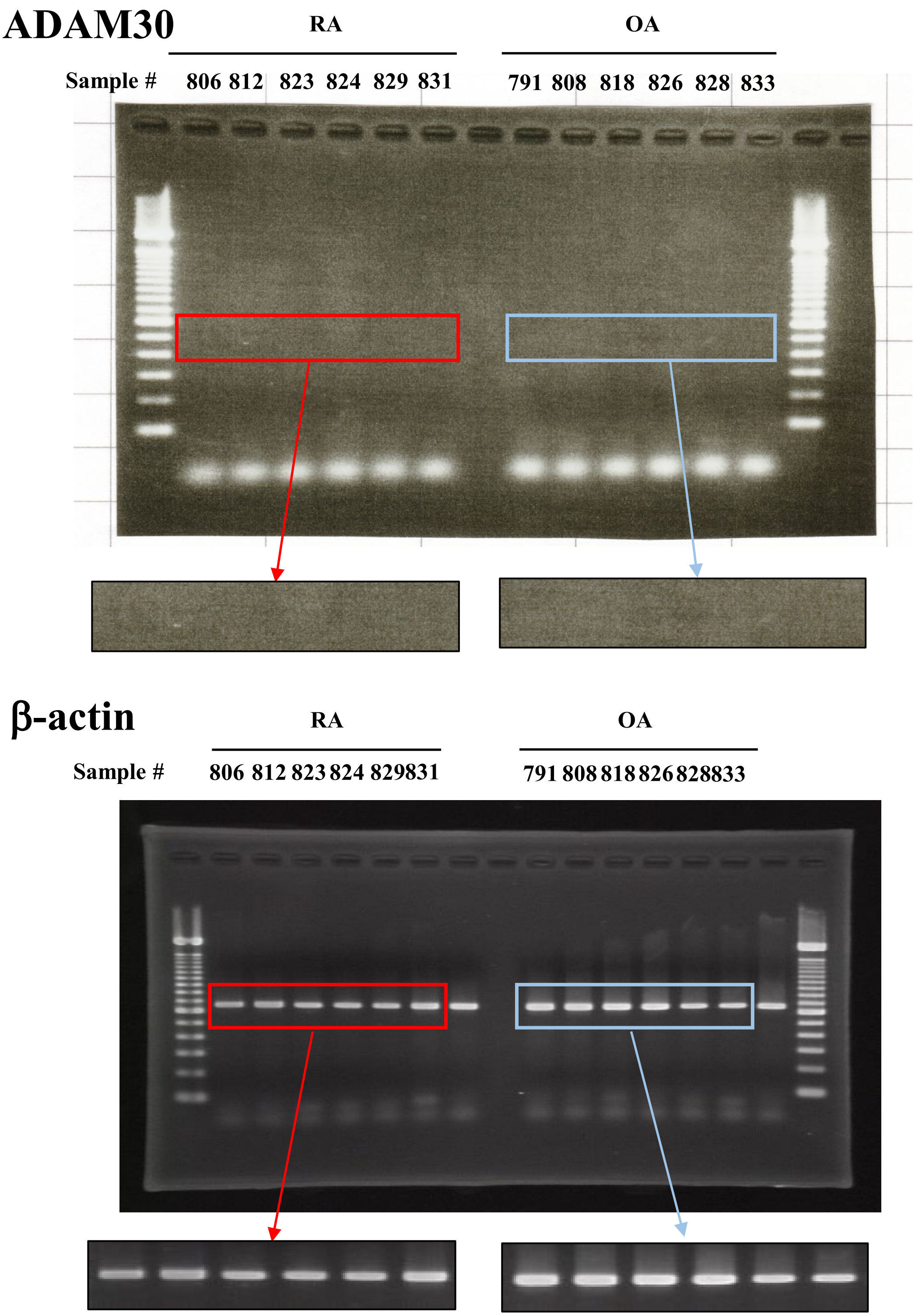

Full-length gels corresponding to ADAM30 and β-actin in the corrected version of Figure 2. Images of ADAM30 and β-actin were obtained by scanning the images mounted on the notebook and from the electronic file, respectively. Adobe Photoshop was used to crop the indicated areas/bands and the areas/bands were placed in Figure 2.

Full-length gels for the corrected version of Figure 2

ADAM species

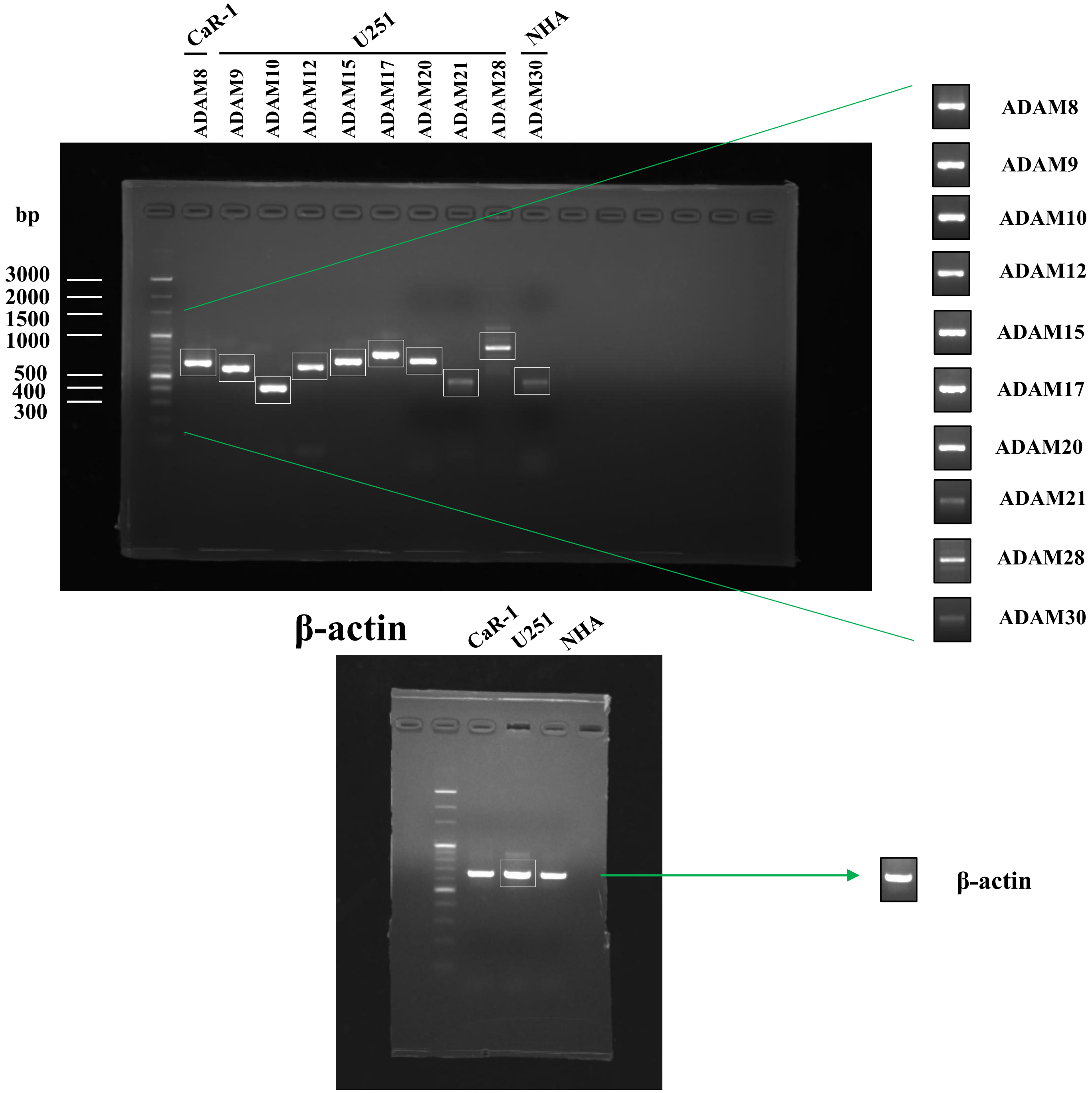

Full-length gels corresponding to control ADAM species and β-actin in the corrected version of Figure 2. As the original data could not be found, we repeated RT-PCR using CaR-1 (human rectal carcinoma cells; JCRB 0207; Health Science Research Resources Bank, Osaka, Japan), U251 (human glioblastoma cells; American Type Culture Collection; Manassas, VA) and NHA (normal human astrocytes; Lonza; Walkersville, MD) cells. Adobe Photoshop was used to crop the indicated bands and the bands were placed in Figure 2.
